# Genetic Databases and Gene Editing Tools for Enhancing Crop Resistance against Abiotic Stress

**DOI:** 10.3390/biology12111400

**Published:** 2023-11-03

**Authors:** Alpana Joshi, Seo-Yeon Yang, Hyung-Geun Song, Jiho Min, Ji-Hoon Lee

**Affiliations:** 1 Department of Bioenvironmental Chemistry, Jeonbuk National University, 567 Baekje-daero, Deokjin-gu, Jeonju 54896, Republic of Korea; joshi.alpana@gmail.com; 2 Department of Agriculture Technology & Agri-Informatics, Shobhit Institute of Engineering & Technology, Meerut 250110, India; 3 Department of Agricultural Chemistry, Jeonbuk National University, Jeonju 54896, Republic of Korea; dustj0501@gmail.com (S.-Y.Y.); vkfkd1819@naver.com (H.-G.S.); 4 School of Chemical Engineering, Jeonbuk National University, Jeonju 54896, Republic of Korea; jihomin@jbnu.ac.kr; 5 Institute of Agricultural Science & Technology, Jeonbuk National University, Jeonju 54896, Republic of Korea

**Keywords:** abiotic stress, CRISPR/Cas9, genome editing, genome databases

## Abstract

**Simple Summary:**

Plants are subjected to various environmental stresses that negatively impact growth and development and limit crop productivity. Therefore, in order to meet the requirements of the growing world population and food security, it is essential to develop cultivars resistant to abiotic stresses. In recent years, with the availability of genetic databases and the advancement in genome editing techniques, it is feasible to edit target genes with precision and create new opportunities for crop improvement that conventional breeding methods could not achieve. The genome-editing method using CRISPR-Cas systems is very powerful and confers exceptional versatility to develop improved cultivars at abiotic stresses. These efficient gene editing techniques facilitate the cultivation of superior-performing genotypes in challenging environmental conditions without compromising yield.

**Abstract:**

Abiotic stresses extensively reduce agricultural crop production globally. Traditional breeding technology has been the fundamental approach used to cope with abiotic stresses. The development of gene editing technology for modifying genes responsible for the stresses and the related genetic networks has established the foundation for sustainable agriculture against environmental stress. Integrated approaches based on functional genomics and transcriptomics are now expanding the opportunities to elucidate the molecular mechanisms underlying abiotic stress responses. This review summarizes some of the features and weblinks of plant genome databases related to abiotic stress genes utilized for improving crops. The gene-editing tool based on clustered, regularly interspaced short palindromic repeats (CRISPR)/CRISPR-associated protein 9 (Cas9) has revolutionized stress tolerance research due to its simplicity, versatility, adaptability, flexibility, and broader applications. However, off-target and low cleavage efficiency hinder the successful application of CRISPR/Cas systems. Computational tools have been developed for designing highly competent gRNA with better cleavage efficiency. This powerful genome editing tool offers tremendous crop improvement opportunities, overcoming conventional breeding techniques’ shortcomings. Furthermore, we also discuss the mechanistic insights of the CRISPR/Cas9-based genome editing technology. This review focused on the current advances in understanding plant species’ abiotic stress response mechanism and applying the CRISPR/Cas system genome editing technology to develop crop resilience against drought, salinity, temperature, heavy metals, and herbicides.

## 1. Introduction

Agricultural production faces global challenges due to climate change, insufficient arable land, population growth, and abiotic and biotic stresses. Abiotic stress adversely impacts plant growth and development by hampering essential biochemical and physiological activities [1,2]. Climate change, such as extreme temperature, drought, water logging, flooding, and increased soil salinity, adversely affect global agricultural crop production. The production of excessive greenhouse gases is the most significant contributor to global climate change, resulting in intense drought, infrequent rain, and high temperatures. Drought stress negatively impacts crop plants by modifying physiological and biochemical processes such as plant growth habit and duration, metabolism, and resistance mechanisms [3,4,5]. Salinity stress is one of the significant constraints to crop production because it causes oxidative stress due to the generation of reactive oxygen species (ROS) that damage plant cells [6,7]. Similarly, plants respond to heat stress by producing reactive oxygen species (ROS), heat shock proteins (HSPs), and genes encoding scavenger proteins [8,9]. Temperature stress strongly impacts grain filling, leading to poor grain yield [5,10]. In addition, overusing chemical fertilizers/herbicides contaminates agricultural fields with heavy metals such as arsenic (As), copper (Cu), cobalt (Co), cadmium (Cd), iron (Fe), manganese (Mn), nickel (Ni) zinc (Zn), mercury (Hg), lead (Pb) accumulating inside plants, reducing grain fullness, and increasing risk to human and animal health. Arsenic causes a loss of functionality in plant cells, and cadmium inhibits plant growth, as evidenced by stunted plant growth, a decrease in leaf size, shoot growth, and root dry matter [11,12,13,14]. Plants have evolved several defense mechanisms to withstand stress by activating stress-responsive genes via secondary messengers and finally activating several stress-responsive genes and their products [15].

Conventional breeding approaches, including cross-breeding and mutation breeding, have enhanced crop performance under climate change scenarios. However, even with marker-assisted selection, breeding programs can be time-consuming and labor-intensive [16]. Therefore, more efficient and advanced technologies with immediate effects are needed to overcome the drawbacks of traditional breeding methods. The availability of genome sequence data of numerous crop plants and precise genome editing tools has revolutionized plant breeding programs. By genome editing tools, desired changes are possible within the DNAs by the formation of an insertion/deletion (indel) and mutation in the sequences of particular genes via recruiting specific nucleases such as zinc-finger nucleases (ZFNs) and transcription activator-like effector nucleases (TALENs) or Clustered regularly interspaced palindromic repeats (CRISPR)/CRISPR-associated protein 9 (Cas9) system [17]. CRISPR/Cas comprises single-guide RNA (sgRNA) and RNA-guided Cas endonuclease that protects bacteria and archaea from being invaded by mobile genetic elements and bacteriophages [18]. During the process of genome editing, sgRNA recruits Cas endonuclease to a specific site of the genome to catalyze a DNA double-stranded break (DSB), which can be repaired by various DNA repair mechanisms, including non-homologous end joining (NHEJ), homology-directed repairs (HDR), and microhomology-mediated end joining [MMEJ], leading to gene knockout, DNA fragment insertion, deletion, and replacement [19,20,21,22]. Different Cas enzymes recognize Protospacer-Adjacent Motif (PAM) sequences and show unique specificity [23]. Moreover, the gRNA spacer sequence could be readily programmed using online tools to target DNA sites with PAM. The online computational tools also minimize off-target effect (guide specificity) and maximize on-target effect (guide efficiency) by avoiding gRNA sequences showing significant homology with genomic loci at multiple sites [24].

CRISPR technology has revolutionized life science research since it was first applied in 2012. CRISPR–Cas9 and CRISPR–Cpf1 are plants’ best-studied and most widely used CRISPR systems [25,26,27]. In addition, Cas12a and Cas12b systems were also developed for plant genome editing [28]. The applications of CRISPR/Cas9 system in plants have been found in studies about *Nicotiana benthamiana* [29], *Nicotiana tabacum* [30], *Arabidopsis thaliana* [31], *Zea mays* [32], *Oryza sativa* [33], *Triticum aestivum* [34], *Hordeum vulgare* [35], *Setaria italica* [36], *Lycopersicon esculentum* [37], *Solanum tuberosum* [38], *Capsicum annuum* [39], *Brassica napus* [40] *Glycine max* [41], and *Saccharum* spp. [42]. This review summarizes plant genome databases related to abiotic stress and the potential for genome editing technology applications using the CRISPR/Cas9 system in managing abiotic stresses such as drought, salinity, temperature, environmental pollutants, and heavy metal toxicity in important agricultural crop species.

## 2. Genome Databases of Abiotic Stress Gene

Genome databases dedicated to plant abiotic stress genes and genome data provide helpful information on essential plant species. The availability of a genome database of agronomically important plant species facilitates targeted genome modification using gene editing tools, which offers tremendous opportunities to develop improved cultivars with higher yield and resistance to abiotic stress. The publicly available specialized bioinformatics database resources contain valuable information on plant stress genes, such as PlantStress, Plant Stress Gene, Plant Stress Proteome (PlantPReS), Plant miRNA ENcyclopedia (PmiREN), Network-based Rice Expression Analysis (NetREx), PncStress, and Pearl Millet Drought Transcriptome (PMDTDb) databases.

### 2.1. PlantStress

The PlantStress (https://plantstress.com/, accessed on 31 October 2023) website was launched in the year 2000 to serve as a web-based information resource for a meeting place and a consultation facility, and a source of professional updates on the most important issues of plant environmental abiotic stress, including drought, salinity, heat, mineral deficiency, oxidative stress, cold, water logging, and stress combination [43].

### 2.2. Plant Stress Gene Database

The Plant Stress Gene Database (http://ccbb.jnu.ac.in/stressgenes/, accessed on 23 August 2023) contains 259 stress-related genes from different plant species (*Arabidopsis thaliana*, *Arachis hypogaea*, *Glycine max*, *Hordeum vulgare*, *Oryza sativa*, *Pennisetum glaucum*, *Phaseolus vulgaris*, *Saccharum officinarum*, *Lycopersicon esculentum*, *Triticum aestivum*, and *Zea mays*). Additionally, this database offers details on paralogous or orthologous proteins encoded by stress-related genes [44].

### 2.3. Plant Stress Proteome Database (PlantPReS)

PlantPReS (http://www.proteome.ir/, accessed on 23 August 2023) contains a database of 10,600 unique stress-responsive proteins and 20,413 entries from 456 research articles [45]. It is an open online proteomic database comprising >35,086 entries from 577 manually curated articles containing >10,600 unique stress-responsive proteins. A customized BLAST tool has been made available, which is helpful in retrieving the homologous sequences from the database. The interface is user-friendly and features several analysis tools such as a gene ontology, cross-referencing, and information on the expression patterns of stress-responsive proteins.

### 2.4. Plant miRNA ENcyclopedia (PmiREN)

PmiREN (Plant miRNA Encyclopedia) is a functional plant miRNA database available at https://www.pmiren.com/, accessed on 23 August 2023. PmiREN contains 38,186 miRNA loci belonging to 7838 families, 141,327 predicted miRNA-target pairs and phylogenetic trees of conserved miRNA families in 179 species spanning from chlorophytes to angiosperms. It also provides tools for in-depth data mining. Additionally, 116 PARE-Seq libraries were utilized to confirm predicted miRNA-target pairs, and 2331 fully sequenced small RNA libraries were used to quantify miRNA expression patterns [46,47].

### 2.5. Network-Based Rice Expression Analysis (NetREx)

Network-based Rice Expression Analysis server (NetREx) provides information on the expression and interaction of rice genes under various environmental stress and hormonal treatment conditions. NetREx is a web-based server available at https://bioinf.iiit.ac.in/netrex/index.html, accessed on 23 August 2023. It offers a choice of interactable data viewers and modules for analyzing genes under drought, flood, cold conditions, and hormonal treatments (abscisic and jasmonic acid). The server can also explore the expression fold change, gene annotations, and gene pathway analysis. This web server also allows the search of orthologous genes from *A. thaliana*, *T. aestivum*, *Z. mays*, *H. vulgare*, and *S. bicolor* [48].

### 2.6. PncStress

PncStress (https://bis.zju.edu.cn/pncstress/, accessed on 23 August 2023) is a manually curated database of circRNAs, lncRNAs, and microRNAs related to plants’ abiotic and biotic stress conditions. PncStress contains 4227 entries, with 114 species responding to abiotic and biotic stresses. PncStress contains the database of the following plant species, including *Arabidopsis thaliana*, *Brassica* sp., *Gossypium* sp., *Hordeum vulgare*, *Oryza sativa*, *Solanum lycopersicum*, *Solanum tuberosum*, *Triticum aestivum*, *Vitis vinifera*, and *Zea mays*. These entries include 2523 miRNAs, 444 lncRNAs, and 52 circRNAs verified through various experimental techniques [49].

### 2.7. Pearl Millet Drought Transcriptome Database (PMDTDb)

PMDTDb (Pearl Millet Drought Transcriptome Database) is the database of the drought transcriptome of pearl millet available at http://webtom.cabgrid.res.in/pmdtdb/, accessed on 23 August 2023. It catalogs the differentially expressed genes in leaf and root tissues of millet in response to drought, along with transcription factors (TFs), gene regulatory networks (GRNs) with hub genes, and discovery of putative gene region markers such as simple sequence repeats (SSRs), single nucleotide polymorphism (SNP) and, InDels (insertions and deletions). This database is based on a 3-tier architecture developed in PHP and MySQL [50].

## 3. Functional Genomic Approaches and Abiotic Stress Tolerance

Functional genomics approaches have been employed to understand the precise regulatory gene networks associated with complex abiotic stress responses, benefiting crop improvement programs. Several stress-related genes/pathways and regulatory networks have been worked out in the past decades using various functional genomic approaches, including expressed sequence tags (ESTs), transcriptome analysis, and targeted random mutagenesis. Recently advanced sequencing technologies have provided a cost-effective and high-throughput method for generating DNA/RNA sequence data, facilitating the identification of genes/transcription factors mediating stress tolerance. The identified genes can be successfully used in the programs for crop improvement by employing a transgenic method or exploiting genetic variation indicating the trait of interest. Additionally, functional validation of stress-related genes may facilitate unraveling the stress-tolerance networks and designing different functional markers for crop improvement programs.

### 3.1. Sequencing-Based Approaches

The single-pass sequencing of cDNA clones generates partial gene coding sequences, which are the Expressed Sequence Tags (ESTs) [51]. EST databases are efficient tools for discovering genes, comparing interspecies sequences, and providing markers for physical and genetic mapping and clones for expression analysis. Functional genomics studies utilize ESTs due to their quick and cost-effectiveness compared to the whole genome sequencing method. Currently, over a million ESTs of different crop species are available in the EST database at the National Center for Biotechnological Information (NCBI) (http://www.ncbi.nlm.nih.gov/dbEST/, accessed on 31 October 2023), which serves as the reservoir of differentially expressed genes. Additionally, the relative abundance of cDNA libraries created from various plant species and organs under various physiological situations offers early insights into the expression patterns for the more abundant transcripts [15]. Several functional genomics studies have been performed to identify the abiotic stress-responsive transcripts using EST sequencing, Serial analysis of gene expression (SAGE), Super serial analysis of gene expression (SuperSAGE), and massively parallel signature sequencing (MPPS). Plant cDNA libraries were screened for identifying genes involved in abiotic stress response. Screening of the cDNA library from a salt-tolerant rice genotype showed differential expression of two genes in response to multiple stresses [52]. In *Cicer arietinum* (Chickpea), to identify differentially expressed genes in drought-tolerant and -susceptible genotypes, 5494 high-quality drought-responsive EST sequence was generated by suppression subtraction hybridization (SSH) [53].

The SAGE is a highly competent technology that isolates unique sequence tags from individual mRNAs for transcriptome research. Although SAGE is not widely utilized in plants, it is more sensitive than EST in detecting rarely expressed transcripts [54]. SAGE helps to identify a set of specific genes to the cellular conditions and study the gene expression profile of a particular type of cells or organs. By transcriptome analysis using SuperSAGE and high-throughput sequencing, 17,493 SAGE UniTags have been generated from the roots of the drought-tolerant *C. arietinum* variety [55]. Another tag-based method, massively parallel signature sequencing (MPSS), is an open-ended platform for conducting in-depth expression profiling [56]. MPSS allows the identification of millions of signatures per experiment, surpassing even the most extensive SAGE applications, covering hundreds of thousands of tags. Due to more extended tags and high-throughput analysis, MPSS identifies genes with greater specificity and sensitivity [57]. Plant MPSS databases contain publicly available MPSS expression data for many plant species, including *A. thaliana*, rice, grape, *Z. mays*, and soybean [58]. MPSS databases quantify the absolute expression level of most genes. It also provides information about novel transcripts, including regulatory intergenic transcripts, alternative splice isoforms, and antisense transcripts [59].

### 3.2. Genome-Wide Association Studies (GWAS)

Genome-wide association studies (GWAS) have emerged as a powerful tool in identifying variations at the DNA level related to stress tolerance. GWAS provides a robust and potent tool successfully applied in germplasm collections that identifies the regulatory loci associated with resistant phenotypic traits [51]. The two major approaches were used to study the interaction between genotype and phenotype (trait) based on mapping populations, including linkage mapping and association mapping [60,61,62,63,64,65,66]. GWAS can be effectively used for fine genome-wide mapping and also enables finding a higher diversity of alleles at the corresponding loci, which exploits historical recombination events in a population, leading to a better association between the marker and the phenotype with a desired trait. GWAS has identified 155 significant SNPs and 275 genes associated with salt sensitivity in *O. sativa* [67]. Combining QTL mapping and GWAS with transcriptome profiling complements the identification of differentially expressed candidate genes in various crop species. GWAS combination with Meta-QTL analysis can be used to investigate the critical genomic regions and major quantitative traits in *T aestivum* [68,69]. In *T. aestivum*, meta-analysis was performed using previously identified QTLs associated with abiotic stress, including drought, heat, salinity, temperature, and aluminum stress, resulting in 76 meta-QTLs verified using genome-wide association studies [70]. In *Z. mays*, 86 candidate genes and 5 SNPs related to salt tolerance were identified by GWAS [71]. Phosphorus (P) is the essential macronutrient in crop growth and production, and its deficiency is one of the major limiting factors in *G. max* production, especially at the reproductive stage. GWAS and a combination of high-density SoySNP analysis identified 27 P-efficiency-related single nucleotide polymorphisms (SNPs), which can be utilized in breeding high-P-efficiency varieties of *G. max* [72]. GWAS has been used in detecting genetic variations underlying diverse, complex traits in barley, such as cadmium stress [73], cold tolerance [74], drought tolerance [75,76], aluminum tolerance [77,78], and salinity tolerance [79,80]. Heat stress caused a significant decrease in grain nutrient content in *C. arietinum*. GWAS revealed that grain yield negatively correlated with Fe and Zn content and non-significantly with protein content. In *C. arietinum*, 181 marker-trait associated with grain nutrient content (Fe, Zn, and protein) under drought and stress conditions was identified using GWAS [81]. Similarly, GWAS revealed SNPs associated with QTLs involved in drought stress by evaluating the drought tolerance ability of horsegram (*Macrotyloma uniflorum*) germplasms [82].

## 4. Mechanisms of CRISPR/CAS9 Genome Editing

The CRISPR/Cas system relies on an adaptive immune system found in the genomes of bacteria and archaea to protect against the invasion of foreign plasmids or viral DNA [30]. CRISPR/Cas9 is composed of two components of Cas9 and a single guide RNA (sgRNA). The CRISPR/Cas system can be divided into two classes based on the structure and functions of Cas-proteins: Class I (type I, III, and IV) and Class II (type II, V, and VI) [83]. Class I systems contain multi-subunit protein complexes, whereas Class II systems include single effector proteins. Due to its relatively simple structure, type II CRISPR/Cas9 has been well-studied and widely used in genetic engineering. The first Cas protein (Cas9) used in genome editing was originated from *Streptococcus pyogenes* (SpCas9). The Cas9 is a large multi-domain DNA endonuclease, composed of 1368 amino acids, that cleaves the target DNA to generate a double-strand break (DSB) [84]. The genome editing mechanism of CRISPR/Cas9 is generally divided into three steps: recognition, cleavage, and repair. The designed sgRNA guides Cas9 and identifies the target sequence within the interest gene through its complementary base pair of 5′crRNA. As a component of the sgRNA/Cas9 complex, 20 nucleotides at the 5’ end of a sgRNA bind to the target site. Without sgRNA, the Cas9 protein remains inactive. The Cas9 nuclease-induced double-strand break (DSB) is formed at a three-base pair upstream site to the protospacer adjacent motif (PAM). This specific target location should be immediately at the upstream of PAM, a conserved DNA sequence downstream of the cleavage site. The most commonly used nuclease in the genome-editing tool, Cas9 protein recognizes the PAM sequence at 5ʹ-NGG-3ʹ. Several studies have been conducted to improve the efficiency of the CRISPR/Cas system. For example, plants’ unique PAM sites (NGG and NG) were discovered using SpCas9-NG and Cas9-NG variants [85]. SpCas9 orthologues have been recognized from *Streptococcus thermophiles* (St1Cas9) [86] and *Staphylococcus aureus* (SaCas9) [87]. Engineered SaCas9 has been developed to target plant genomic loci with the PAM sequence of NNNRRT [88]. Additionally, engineered SpCas9 has been developed to amend plant genomic loci with PAM sequences of NGG, NG, NRNH, NGN, NRN, or NYN [89,90,91,92,93,94,95] [Table 1].

**Figure 1:**
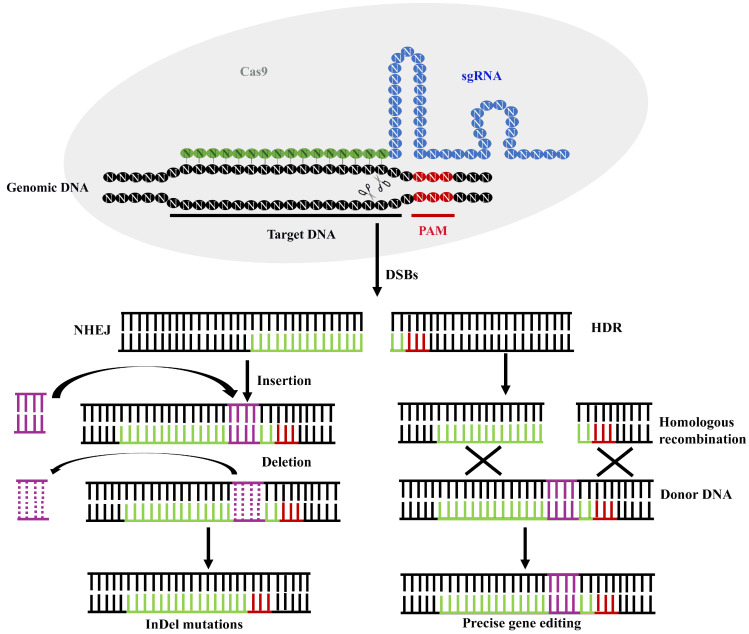
Schematic illustration of the mechanism of genome editing using CRISPR/Cas9 system. The single guide RNA (sgRNA)/CRISPR-associated protein 9 (Cas9) complex binds to the target site at a complementary region of the genomic DNA. The protospacer adjacent motif (PAM) is recognized by Cas9 nuclease, which introduces the double-stranded breaks (DSBs) within the target DNA at a site three base pair upstream to the PAM. Double-stranded breaks (DSBs) of the target DNA are repaired by non-homologous end joining (NHEJ), resulting InDel mutations (insertion or deletion) or homology-directed repair (HDR) in the presence of a donor DNA, resulting in precise gene editing.

Two mechanisms repair DSBs created by Cas9 protein: homology-directed repair (HDR) and non-homologous end joining (NHEJ) (Figure 1). The HDR is exact and requires a homologous DNA donor template with a target sequence. The HDR mechanism is primarily active in the G2 and late S phases of the cell cycle. HDR executes the specific gene insertion or gene replacement by adding a donor DNA template with sequence homology at the predicted DSB site [21]. The NHEJ is the leading and most efficient cellular repair mechanism and is active in all cell cycle phases. Unlike HDR, NHEJ is an error-prone mechanism that may result in indels (short insertions and deletions) at the cleavage site, leading to frameshift mutation or premature stop codon in the sequence. NHEJ accelerates the repairs created by DSBs by joining the cleaved DNAs through an enzymatic activity in the absence of exogenous homologous DNA [19,20,22].

Targeted gene modifications were performed using the Cas9-gRNA system in multiple plant species, which suggests the system can function in different organisms [29,30,31,32,33,34,37,38,39,41,42]. Two main criteria for CRISPR/Cas genome editing are efficacy and specificity. Numerous computational in silico tools have been developed for designing guide RNAs to predict cleavage efficiency and accurate target specificity (Table 2). Online computational tools also facilitate the design of specific guide RNAs for particular genes of interest to avoid potential off-target effects. The freely available online tools for sgRNA design and quality check are CHOPCHOP (https://chopchop.cbu.uib.no/, accessed on 23 August 2023) [103], Cas-OFFinder (http://www.rgenome.net/cas-offinder/, accessed on 23 August 2023) [104], CCTop (https://cctop.cos.uni-heidelberg.de/, accessed on 23 August 2023) [105], CRISTA (https://crista.tau.ac.il/, accessed on 23 August 2023) [106], CRISPR-GE (http://skl.scau.edu.cn/, accessed on 23 August 2023) [107], CRISPR-P (http://crispr.hzau.edu.cn/CRISPR2/, accessed on 23 August 2023) [108], CRISPR-PLANT V2 (http://omap.org/crispr2/, accessed on 23 August 2023) [109], CRISPRlnc (http://www.crisprlnc.org/, accessed on 23 August 2023) [110], SNP-CRISPR (https://www.flyrnai.org/tools/snp_crispr/web/, accessed on 23 August 2023) [111], and PnB Designer (https://fgcz-shiny.uzh.ch/PnBDesigner/, accessed on 23 August 2023) [112].

## 5. Impact of CRISPR/Cas9-Based Genome Editing on Abiotic Stress Tolerance

Due to abiotic stresses, plant growth and development are affected, which can cause crop yield reduction at approximately 50% [113]. Though productivity increases to a large extent by traditional breeding, it may cause a loss of genetic variety and fitness. In addition to the development time period, it relies on natural allelic variants, which makes it challenging to develop the desired characteristic and to ensure the sustainability of production. Genome editing must include precise modifications at specific sites to perform desired changes to the DNA sequence [20,21,30]. Therefore, genome-editing techniques employing sequence-specific nucleases (SSNs) have become popular in plant research to develop improved cultivars in terms of yield, nutrition content, and resistance to environmental stresses. The SSNs introduce DNA DSBs at a target site, stimulating the cellular DNA repair and resulting in genome alterations, including targeted mutagenesis, gene insertion, and gene replacement [114]. In recent years, three types of genome-editing techniques have been widely used, such as zinc finger nucleases (ZFNs), transcription activator-like effector nucleases (TALENs), and clustered regularly interspaced palindromic repeat CRISPR/Cas protein systems. Due to complex procedures and high failure rates, ZFN and TALEN have not been utilized extensively, whereas CRISPR/Cas was successfully used in various crop improvement programs. CRISPR/Cas9-based genome editing is accurate because it targets specific sites of particular genes involved in stress response pathways and modifies genes to develop plants’ ability to withstand environmental stress [30,115]. Additionally, the CRISPR/Cas system has been used to introduce critical agricultural traits, including plant resistance against abiotic and biotic stress, and other agronomically important traits (increased grain size and grain weight) into many economically important crops, such as *O. sativa*, *T. aestivum*, *Z. mays*, *L. esculentum*, *S. tuberosum*, *N. tabacum*, *Gossypium* spp., *G. max*, *Brassica* sp., *S. italica*, and *Saccharum* spp. [5,30,31,32,33,34,37,39,40].

### 5.1. Improvement in Drought Stress Tolerance using CRISPR/Cas System

Drought stress can reduce crop yields by 50–70% in different crops due to significant reductions in plant growth and development. For example, a 27–40% yield reduction has been observed in *C. arietinum*, 42% in *G. max*, 50% in *O. sativa*, 21% in *T. aestivum*, 68% in *V. unguiculata,* and 40% in *Z. mays* [5]. Plants experience morphological, physiological, biochemical, and molecular changes in response to drought stress. CRISPR/Cas technique was successfully applied to enhance the drought resistance of rice crops by modifying the expression of drought and other stress-related genes [116]. The potential of CRISPR/Cas gene editing has been documented in various crop species against drought stress (Table 3). Researchers aimed to enhance plants’ ability to withstand drought stress and reduce crop losses by altering drought-related genes. A truncated version of gRNAs (<20 nucleotides) with target sequences in plant cells was used to improve the specificity of CRISPR/Cas9 and eventually generate altered alleles for *OST2* (Open Stomata 2). The novel mutant alleles for *OST2* exhibited drought tolerance by altered stomatal closing in response to environmental stress in *A. thaliana* [117]. Similarly, the remodeled CRISPR/Cas9 activation system activates vacuolar H+-pyro phosphatase *AVP1*, leading to an increase in single-leaf area, an increase in leaf numbers, and an enhancement of stress tolerance to drought [3]. Improved drought resistance was found in homozygous CRISPR/Cas9-edited *MIR169a* T3 plants using a combinatory dual-sgRNA/Cas9 vector containing deleted miRNA gene regions (*MIR169a* and *MIR827a*) [118]. Histone acetyltransferase (*HAT*) modifies chromatin histone, exposing DNA to the transcriptional machinery and regulating gene expression. Stable transgenic plants expressing chimeric *dCas9HAT* in *A. thaliana* showed higher chlorophyll content, faster stomatal aperture, and an improved survival rate under drought-stress conditions [119]. *Trehalase* (*TRE1*) gene silencing through the use of the CRISPR/Cas9 system developed drought tolerance in *A. thaliana* [120]. Transcriptome analysis using microarray technology is the best technique that has proven helpful in discovering many stress-inducible genes/stress-inducible transcription factors including the DRE-binding protein (*DREB*) members, ethylene-responsible element binding factor (*ERF*), zinc-finger, *WRKY*, *MYB*, basic helix-loop-helix (*bHLH*), basic-domain leucine zipper (*bZIP*), *NAC* (*NAM*, *ATAF1*, and *CUC2*), and homeodomain transcription factor families [4]. Overexpressing *AtNAC07*, *AtNAC019*, and *AtNAC055* can enhance tolerance to drought in *A. thaliana* [121]. Dehydration-responsive element binding (*DREB*) proteins are one of the most prominent transcription factors and have a significant role in signaling networks regulating various plant development processes and stress responses. The overexpression of *DREB1A/CBF3* (C-repeat binding factor) under the stress-inducible RD29A promoter improved drought tolerance in transgenic *T. aestivum* [122]. Drought tolerance in *T. aestivum* was enhanced by altering Dehydration-responsive element binding 2 (*TaDREB2*) and Ethylene Responsive Factor 3 (*TaERF3*) using the CRISPR/Cas system [123].

Abscisic acid (ABA) plays a vital role in drought tolerance by regulating the expression of many drought-related genes. ABA regulates the expression of genes through ABA-responsive element (*ABRE*) binding protein/ABRE binding factor (*AREB/ABF*). Drought stress tolerance has been demonstrated by over-expression of *AREB1*, as compared to the *AREB1* knockout mutant [124]. In *A. thaliana*, *ABF1*, *ABF3*, *AREB1/ABF2*, and *AREB2/ABF4* are expressed in response to ABA and drought stress in vegetative tissues, whereas *ABI5*, *AREB3*, *DPBF2,* and *EEL* are expressed during seed maturation [124,125,126]. Abscisic acid (ABA) signaling is regulated by ABA-induced transcription repressors (*AITRs*). The CRISPR/Cas9 system was used in soybean (*Glycine max*) to target the six *GmAITR* genes and generated Cas9-free *gmaitr36* double and *gmaitr23456* quintuple mutants, enhancing salinity tolerance [41]. Similarly, the Dehydration-responsive element [*DREB1*]/*CBF* is responsible for the ABA-independent induction of several genes in response to osmotic and cold stress, for example, *RD29A/COR78/LTI78* gene in *A. thaliana.* The lateral organ boundaries domain (*LBD*) genes play essential roles in lateral organ development. CRISPR/Cas9 knockout of *SlLBD40* improved drought tolerance in *L. esculentum* compared with overexpressing transgenic and wild-type plants [127]. Mitogen-activated protein kinases (*MAPKs*) are important signaling molecules that respond to drought stress. Similarly, the CRISPR-Cas knockout mutant for the *SlMAPK3* gene down-regulated the expressions of drought stress-responsive genes: *SlLOX*, *SlGST*, and *SlDREB* [128,129]. The CRISPR-Cas9 mediated *dst^∆184–305^* mutation in the *DST* (drought and salt tolerance) gene of *O. indica* cv. *MTU1010* produced mutants having broader leaves and reduced stomatal density, resulting in improved leaf water retention under drought stress [130]. The SNF1-related protein kinase 2 (*SnRK2*) is the primary regulator of hyper-osmotic stress signaling and abscisic acid (ABA)-dependent plant development. A knockout mutant of the *SAPK2* gene improved drought tolerance in *O. sativa* by affecting ABA signaling [131]. The CRISPR/Cas9-mediated knockout of *SRL1* and *SRL2* (Semi-rolled leaf 1, 2) and *ERA1* (Enhanced Response to ABA1) genes improved drought tolerance in *O. sativa*. *OSERA1* mutant lines display similar leaf growth as wild-type plants but enhanced primary root growth [132]. The *SRL1* and *SRL12* knockout mutants had fewer stomata, a slower rate of transpiration, less chlorophyll, vascular bundles, and rolled leaves than the wild type [133]. Plant *ITPKs* (Inositol trisphosphate 5/6 kinases) participate in abiotic stress signaling, and the *itpk1* mutant created using programmable nuclease Cas9 displayed higher tolerance to salinity stress than deletion mutants in *H. vulgare* [134]. In *B. napus*, the *bnaa6.rga* mutant generated through CRISPR/Cas9 showed enhanced tolerance to drought stress by promoting stomatal closure through increased ABA sensitivity [40].

*ARGOS* is a negative regulator of the ethylene response, and CRISPR/Cas9-mediated editing of the ethylene response factor *ARGOS8* improved drought tolerance in *Z. mays* [135]. *WRKY* are plant-specific transcription factors that play essential roles in abiotic stress response. Several *WRKY* transcription factors were identified in plant species, including *A. thaliana*, *O. sativa*, *G. max*, *T. aestivum*, and *H. vulgare* [136,137,138]. Overexpression of *ZmWRKY40* promoted root growth and reduced the water loss rates in transgenic *A. thaliana* under drought stress [137]. Overexpression of the *T. aestivum TaWRKY33* enhanced the drought and heat tolerance in transgenic *A. thaliana* [136]. *OsWRKY5* is expressed in developing leaves at the seedling and heading stages of *O. sativa*. It is the negative regulator of drought, and its expression was reduced under drought stress and by treatment with NaCl, mannitol, and abscisic acid (ABA) [138]. These studies indicated the efficiency of the CRISPR/Cas system in developing drought-tolerant cultivars by knocking out or overexpressing target genes through precise genome editing.

### 5.2. Improvement in Salinity Stress Tolerance Using CRISPR/Cas System

Genome editing and genetic engineering tools have been utilized to target genes involved in ion transport for regulating osmotic adjustment under salt stress. Soil salinity is a critical abiotic stress affecting crop productivity worldwide. Plant salt tolerance is the ultimate manifestation of several physiologic processes, including Na^+^ uptake and exclusion, ionic balance (especially Na^+^/K^+^ ratio), and distribution [6]. Salt Overly Sensitive 1 (SOS1) is an extensively characterized Na+ efflux transporter in *G. max*, *A. thaliana*, and *T. aestivum*. The *gmsos1* mutants were generated using the CRISPR-Cas9 system in *G. max,* and the resulting mutant displays a significant accumulation of Na+ in the roots and increased salt sensitivity [139]. In *A. thaliana*, the *SOS* signal transduction pathway (including *SOS1*, *SOS2*, and *SOS3* genes) is essential for ion homeostasis and salt tolerance. The *SOS1* gene isolated from durum wheat (*T. durum*) conferred salinity tolerance to the *sos1* mutant of *A. thaliana* [140]. Similarly, the CRISPR/Cas9 knockout of the *AITR* family genes (*AITR3* and *AITR4*) in *A. thaliana* enhanced tolerance to drought and salinity stress without fitness costs [7]. A gene cluster containing (*T5G46490*, *AT5G46500*, A*T5G46520*) and (NLRs; *AT5G46510*) is involved in osmotic stress tolerance. CRISPR/Cas9-mediated mutagenesis generated single and double knockout lines for *ACQOS* alleles in *A. thaliana*. *A. thaliana* plants containing complete deletions or pseudogenization-induced polymorphisms in *ACQOS* and *AT5G46510* show considerable tolerance to salt stress, suggesting the role of *ACQOS* in salt stress tolerance [141].

Nitric oxide (NO) plays a vital role in cytoprotection by regulating the level of ROS and inducing transcriptional changes, leading to the modulation of protein function [142]. Reactive oxygen species (ROS) are highly reactive molecules typically produced in response to environmental stress, such as salinity and drought. Histone acetyltransferase *TaHAG1* is a vital regulator to strengthen the salt tolerance of *T. aestivum*. *TaHAG1* contributed to salt tolerance by modulating ROS production and signal specificity. CRISPR-mediated mutagenesis of *TaHAG1* validated the role of *TaHAG1* in salt tolerance in *T. aestivum* [143]. Salt stress increases ROS production and is responsible for oxidative damage, membrane injury, lipid peroxidation (malondialdehyde), and ultimately cell death. CRISPR/Cas9-mediated mutagenesis of the *osbhlh024* gene negatively regulates the functions of Na^+^ and K^+^ transporter genes, suppressing the higher accumulation of MDA and H_2_O_2_, leading to salt tolerance in *O. sativa* [144].

Several quantitative trait loci (QTLs) and genes associated with regulating salt stress tolerance have been identified in *O. sativa,* including the *NHX* family (*OsNHX1*, *OsNHX2*, *OsNHX3*) [145,146,147], *HKT* family (*OsHKT1*, *OsHKT2*, *OsHKT7*) [148,149,150,151], *DCA1* [152], *DST1* [130,153], *OsKAT1* [154], *OsBADH1* [155], *OsNAC5* [156], *OsbZIP71* [157], *SKC1*, *OsHAL3*, *P5CS*, *SNAC2*, *OsNAP*, *OsRRY* [158,159], and *OsSALP1* [113,160]. CRISPR/Cas9-mediated knockout of several salt stress genes significantly improved salinity tolerance in various crops. CRISPR/Cas9 and third-generation hybrid rice system approaches were employed to generate the *OsRR22* mutant, which exhibited enhanced salinity tolerance without any morphological and physiological changes relative to the wild-type [159]. A receptor-like kinase gene *OSBBS1/OsRLCK109* played vital roles in leaf senescence and salt stress response [161]. CRISPR/Cas9-mediated editing of the *SAPK1* and *SAPK2* genes showed resistance to salt stress in *O. sativa* [131]. The mutant alleles of *DST* (drought and salt tolerance) generated using the CRISPR/Cas9 method showed reduced stomatal density by downregulating stomatal developmental genes (*SPCH1*, *MUTE, ICE1*), resulting in a high level of salt tolerance in the seedling stage of *O. sativa* [130]. Argonaute (*AGO*) proteins primarily function in gene silencing by forming RNA-induced silencing complexes. CRISPR/Cas9-mediated *AGO2*-knockout mutant lines showed few morphological changes compared to wild-type rice. The overexpression of *AGO2* under the control of the cauliflower mosaic virus 35S led to a simultaneous increase in salt tolerance and grain length [162]. Transcription factors such as *AP2/ERF*, *NAC* (*NAM*, *ATAF1/2*, *CUC2*), and *WRKY* families induce stress-responsive gene expression in response to environmental signals. APETALA2/ethylene response factor (*AP2/ERF*) plays crucial roles in transcriptional regulation and defense response against biotic and abiotic stress. Editing of the *OsRAV2* (AP2/ERF domain-containing RAV) gene using CRISPR/Cas9 showed tolerance to salt stress [163]. *DOF* transcription factor (DNA-binding with one finger) regulates the elongation of the primary root positively by controlling cell proliferation in the root meristem by restricting ethylene biosynthesis. *O. sativa* mutant *osdof15* showed reduced cell proliferation and primary root elongation in the root meristem [164]. A knockout mutant (*ospqt3*) with CRISPR-Cas9 technology displayed greater resistance to oxidative and salt stress with high expression of *OsGPX1*, *OsAPX1*, and *OsSOD1* [165]. Similarly, CRISPR/Cas9 knockout of *OsmiR535* demonstrated salinity tolerance in *O. sativa* against NaCl, ABA, dehydration, and PEG stresses [166]. *OsNAC45* plays a vital role in ABA signal responses, and overexpression of *NAC45* enhances salt tolerance in *O. sativa*. *OsNAC45* may regulate the expression of seven genes namely *CYP89G1*, *DREB1F*, *EREBP2*, *ERF104*, *PM1*, *SAMDC2*, and *SIK1* [167]. Targeted mutagenesis of the *OsOTS1* gene using the CRISPR/Cas9 system in the *O. sativa* cv. Kitaake enhanced sensitivity to salt with reduced root and shoot biomass, indicating that *OsOTS1* has a major role in salt stress tolerance [168].

Hormones like Gibberellic and Absiscic acid signaling pathways significantly affect salt stress. *OsPIL14-SLR1* (Phytochrome Interacting Factor-Like14–DELLA protein, SLENDER RICE1) controls seedling growth in response to salt stress. CRISPR/Cas9 mediated *ospil14* mutants produce normal mesocotyls and longer roots than wild-type plants [169]. *ZmWRKY114* is a negative regulator of salt-stress responses, and overexpressed *WRKY114* exhibited enhanced salt-stress sensitivity and reduced ABA sensitivity [170]. Salinity stress tolerance was identified in several stress-related genes like *HyPRP1* (Hybrid proline-rich protein 1), *HKT1*, *HKT1* (High-affinity potassium transporter1;2), *RAD51/54* (DNA repair and recombination protein 51/54) and *PR-1* (Pathogenesis-related protein 1) [37,151]. *HyPRP1* is a negative regulator of salt stress responses, and CRISPR-Cas9 mediated genome editing of *HyPRP1* in *L. esculentum* resulted in the elimination of the functional domain of proline-rich protein. Plants carrying such variants, *PR1v1* lacking proline-rich domain, *PR2v2* and *PR2v3* lacking eight cysteine motifs, showed improved germination compared to wild type under osmosis stress [37]. A significant improvement in Homology-directed repair (HDR) using CRISPR/LbCpf1-geminiviral multi-replicons was reported to target marker-free salt-tolerant *HKT1*, *HKT2* alleles in *L. esculentum* [151]. Self-pollinated offspring plants carrying the *HKT1*, and *HKT2* allele showed stable inheritance and germination tolerance under salt stress conditions (100 mm NaCl concentration). In *Z. mays*, Na^+^ Content1 (*ZmNC1*) encodes an HKT-type transporter *ZmHKT1*, preferentially expressed in root stele. CRISPR/Cas9 knockout lines of *ZmHKT1* increase Na^+^ concentration in xylem sap and cause increased root-to-shoot Na^+^ delivery, indicating that *ZmHKT1* promotes leaf Na^+^ exclusion and salt tolerance by withdrawing Na^+^ from the xylem sap [150]. Mutations in genes *OsRR9* and *OsRR10* generated using the CRISPR/Cas9 system enhanced salinity tolerance but reduced panicle and spikelet numbers per panicle in *O. sativa* [171]. CRISPR/Cas9 mediated mutagenesis of the *ARF* (Auxin Response Factors) gene generates a *slarf4* mutant that displayed salinity and drought tolerance in *L. esculentum* by stimulating root development and stomatal function [172] (Table 3). These studies demonstrate the potential role of CRISPR/Cas mutagenesis in knocking out genes responsible for salinity tolerance in plants.

### 5.3. Improvement in Heat Stress Tolerance Using CRISPR/Cas System

Heat stress is the third most crucial abiotic factor that adversely affects the yield and quality of plants during entire growth stages, from germination to harvesting. Plants respond to heat stress in various ways, including alterations in enzymes that generate reactive oxygen species (ROS), heat shock proteins (HSPs), and genes encoding scavenger proteins [8]. The advancement of structural and functional genomics technologies in plants has led to the identification and characterization of various temperature-stress-related genes to enhance plant ability to withstand heat [9]. The heat-shock-induced CRISPR/Cas9-mediated genome editing efficiently produces heritable targeted mutations. In *O. sativa*, a heat-shock-inducible CRISPR/Cas9 system was employed to generate targeted and heritable mutations [173]. Similarly, CRISPR/Cas9-based genome editing targeted the heat-sensitive gene, Slagamous-Like 6 (*SIAGL6*), resulting in increased fruit setting under heat stress conditions in *L. esculentum* [174]. Calcium-dependent protein kinase 28 (*cpk28*) mutant was generated using CRISPR/Cas9 mediated editing and displayed thermotolerance in *L. esculentum* [175]. Brassinazole Resistant 1 (*BZR1*) is involved in thermo-tolerance by regulating the Feronia (FER) homologs. CRISPR/Cas9-based *bzr1* mutant reduced apoplastic reactive oxygen species (H_2_O_2_) production and enhanced heat tolerance *L. esculentum* [176]. Photosynthetic apparatus is highly susceptible to thermal damage. Heat-sensitive albino1 (*hsa1*) mutant harbors a recessive mutation in a gene encoding fructokinase-like protein2 (*FLN2*), resulting in a severe albino phenotype with defects in early chloroplast development. In *O. sativa*, *hsa1* mutants showed increased sensitivity to heat stress but had a faster greening phenotype than wild-type plants [177]. Knockout of the *ZmTMS5* gene of *Z. mays* using the CRISPR/Cas9 system generated homozygous T1 *tms5* thermosensitive male-sterile plants that are male-sterile at 32 °C but are male-fertile at 24 °C [178]. *NCED4* (9-cis-Epoxycarotenoid Dioxygenase4) is a key regulatory enzyme in the biosynthesis of abscisic acid (ABA). Similarly, stable homozygous *NCED4* mutants generated using CRISPR/Cas9 were capable of germinating seeds at a higher temperature (>70% germination at 37°) in Lettuce (*Lactuca sativa*) [179]. Another transcription factor, *OsNAC006*, is regulated by temperature stress, hormones (abscisic acid, indole-3-acetic acid, and gibberellin), NaCl, polyethylene glycol, and reactive oxygen species. Furthermore, CRISPR-Cas9 mediated knockout of *OsNAC006* causes drought and heat sensitivity in *O. sativa* [180] (Table 3). These studies highlight the application of the CRISPR/Cas9 system to target heat-sensitive genes for developing plant resistance against heat stress.

### 5.4. Improvement in Cold Stress Tolerance Using CRISPR/Cas System

Cold stress due to chilling and freezing temperatures hinders plant growth and development. Low temperature inhibited plant metabolic activities, producing osmotic and oxidative stress [5]. Mechanical damage and metabolic dysfunction caused by freezing temperatures reduced plant growth and development. In *A. thaliana*, the two subclasses, namely *DREB1/CBF* and *DREB2,* are induced by cold and dehydration, respectively [4]. Expression of *T. aestivum TaICE41* and *TaICE87* in transgenic *A. thaliana* activated the expression of *COR* genes and consequently led to the enhancement of cold tolerance, but only after cold acclimation [181]. The overexpression of *AtDREB1A* under the RD29A promoter conferred increased drought and freezing tolerance to transgenic *A. thaliana* plants without affecting growth and development [182]. Several studies have demonstrated that *WRKY* transcription factors are essential in cold, heat, drought, and salinity stress [183]. In Cucumber (*Cucumis sativus*), *CsWRKY46* is a *WRKY* transcription factor that confers cold resistance in transgenic plants by controlling cold-stress responsive genes in an ABA-dependent manner. Overexpression of *CsWRKY46* regulates freezing and chilling resistance and increases the expression of stress-inducible genes, including *RD29A* and *COR47* [184]. In strawberries (*Fragaria vesca*), *FvICE1* is a positive regulator of cold and drought resistances, and the overexpressed *FvICE1* gene improved cold and drought tolerance at the phenotypic and physiological levels. Mutant (*fvice1*) generated using the CRISPR/Cas9 system demonstrated lower tolerance to cold and drought. This study indicates the potential of CRISPR/Cas9 system in understanding the function of stress-related genes [185]. *A. thaliana HOS1* (High Expression of Osmotically Responsive Genes 1) is a Ring finger E3 ubiquitin ligase, a key regulator of cold signaling. CRISPR/Cas9-mediated knockout of the *HOS1* gene showed abiotic stress tolerance, accumulation of secondary metabolites, and expression of the biosynthetic genes [10].

The C-repeat binding factors (CBF) are highly conserved CBF cold-response-system components in many plant species. It has a major role in cold acclimation and freezing tolerance in response to low temperatures. CRISPR–Cas9-mediated *SlCBF1* mutagenesis reduced chilling tolerance of *L. esculentum* because of higher electrolyte leakage, increased indole acetic acid contents, decreased abscisic acid, methyl jasmonate, and down-regulated CBF-related genes [186]. Similarly, CRISPR–Cas9-mediated mutagenesis of *CGFS*-type *GRXs* (*SlGRXS14*, *SlGRXS15*, *SlGRXS16*, and *SlGRXS17*) genes showed the sensitivity of *Slgrxs* mutants to various abiotic stresses as compared to wild-type in *L. esculentum* [187]. Plant annexins are Ca2+-dependent phospholipid-binding proteins that play a role in development and protection from environmental stresses. CRISPR/Cas9-mediated knockout mutant of annexin gene *OsAnn3* decreased cold tolerance in *O sativa* [188]. *OsMYB30* confers cold sensitivity by interacting with an *OsJAZ9* protein and downregulating the expression of β-amylase genes in *O. sativa* [189]. Novel mutants were generated by simultaneously editing three genes, *OsPIN5b* (panicle length gene), *GS3* (grain size gene), and *OsMYB30*, using the CRISPR–Cas9 system showed higher yield and excellent cold tolerance [190]. PYR1]/PYR1-like [PYL]/regulatory components of the ABA receptor detects abscisic acid during abiotic stress. CRISPR/Cas9 technology was used to edit *PYL1*–*PYL6* and *PYL12* (group I) and *PYL7*–*PYL11* and *PYL13* (group II) genes of *O. sativa* [191]. A knockout mutant of the *OsPRP1* gene of *O. sativa* generated by CRISPR/Cas9 demonstrated less antioxidant enzyme activity and accumulated lower levels of proline, chlorophyll, abscisic acid (ABA), and ascorbic acid (AsA) content relative to wild-type plants under low-temperature stress [192]. CRISPR/Cas9-mediated base editing technology generated the point mutations in two genes (*OsWSL5* and *OsZEBRA3*) in protoplasts and regenerated plants of *O. sativa*. *OsWSL5* encodes a novel chloroplast-targeted pentatricopeptide repeat protein essential in rice chloroplast biogenesis under cold stress [193,194] (Table 3). These studies indicated the potential of CRISPR/Cas9–mediated mutagenesis in developing resistance to chilling and freezing temperatures in drought-tolerant cultivars by knocking out or overexpressing target genes through precise genome editing.

### 5.5. Improvement in Metal and Herbicide Stress Tolerance Using CRISPR/Cas System

Heavy metals, including arsenic (As), copper (Cu), cobalt (Co), cadmium (Cd), iron (Fe), manganese (Mn), nickel (Ni), zinc (Zn), mercury (Hg), lead (Pb) have accumulated in soils as a result of various human activities such as the overuse of agricultural chemicals (fertilizer, herbicides, and pesticides), improper disposal of industrial and sewage waste [11]. Heavy metals are responsible for causing oxidative stress in plants and generate reactive oxygen species, leading to cellular injury. CRISPR-Cas9-mediated mutagenesis was used to improve heat metal stress in plants. *OXP1* is one of the enzymes involved in 5-oxoproline metabolism and the pathway for glutathione recycling. The oxp1/CRISPR tolerated plants tolerated heavy metals, such as Cd and amisulbrom (a sulfonamide) [13]. Cadmium stress activates the antioxidant defense system and increases the production of abscisic acid (ABA), glutathione (GSH), salicylic acid (SA), jasmonic acid (JA), and nitric oxide (NO) [195,196]. Absorption of Cd by the roots is mediated by *O. sativa* genes (*OsNramp1*, *OsCd1*, and *OsNramp5*). In *O. sativa*, *OsHMA2*, *OsCCX2*, and *CAL1* regulate Cd transport to the xylem, and *OsHMA3* negatively regulates Cd xylem loading. Manipulation in the expression of these genes through CRISPR/Cas9 can minimize the Cd concentration in *O. sativa* [197]. CRISPR/Cas9 knockout mutants of *OsLCT1* and *OsNramp5* exhibited reduced levels of Cd in *O. sativa* [198]. *OsNRAMP1* modulates metal ion and reactive oxygen species (ROS) homeostasis. *Osnramp1* mutants generated through CRISPR/Cas9 displayed reduced levels of heavy metals (Cd and Pb) in leaves and grains of *O. sativa* [14]. The *OsHAK1* gene encodes a Cs^+^-permeable K^+^ transporter that regulates the absorption and translocation of cesium [Cs^+^] in *O. sativa*. CRISPR/Cas9 knockout mutant of *OsHAK1* exhibited a significant reduction in Cs^+^ uptake in *O. sativa* [199]. Potassium [K^+^] is a critical macronutrient for plant growth and development. ROS was strongly induced and accumulated in K^+^-deficient plants. Gene *Prxs* have been involved in the toxic reduction and intracellular H_2_O_2_ scavenging. The overexpressed *OsPRX2* gene improved tolerance to K^+^ deficiency by affecting stomatal movement in *O. sativa* [200]. *OsARM1* (Arsenite-Responsive MYB1) is the R2R3 MYB transcription factor that regulates arsenic-associated transporters genes in *O. sativa*, and the knockout mutant (*osarm1*) generated using CRISPR/Cas system displayed improved tolerance to arsenic [201].

Herbicides destroy weeds and crop plants by interfering with or altering their metabolic processes, resulting in low yields. Thus, tolerance to herbicides can be one of the essential traits for crop plants that could improve farming practices and crop productivity. CRISPR/Cas-based genome editing techniques efficiently modify target genes and hold great potential in engineering plants with herbicide resistance [202]. In recent years, CRISPR-Cas9-based technology has been used to generate herbicide-tolerant crops, including *O. sativa*, *Z. mays*, and *G. max* [203,204,205,206]. Acetolactate synthase (*ALS*) catalyzes the step in the biosynthesis of the branched-chain amino acids, including leucine (Leu), isoleucine (Ile), and valine (Val). Enzyme *ALS* is the target enzyme for two classes of herbicides: sulfonylurea and imidazolinone. Tolerance to *ALS*-inhibiting herbicides has been developed using a genome editing system in *A. thaliana*, *O. sativa*, *T. aestivum*, *Z. mays*, *S. lycopersicon*, and *Saccharum* spp. [42,203,204,205,206,207,208]. Herbicide-resistant plants were generated through CRISPR/Cas9-mediated homologous recombination of *ALS* in *O. sativa* [204]. Similarly, chlorsulfuron resistance was enhanced in *Z. mays* by editing the *ALS2* gene (substitution P165 with Ser) using either single-strand oligonucleotides or double-strand DNA vectors as repair templates [203]. Moreover, P171F substitution in the *OsALS1* allele was performed in the *O. sativa* cultivar Nangeng 46 by precise base editing, resulting in tolerance to the herbicide bispyribac-sodium [208]. Four different missense mutations (P171S, P171A, P171Y, and P171F) in the P171 codon of the *ALS* gene showed different degrees of tolerance towards five typical herbicides (Sulfonylurea, imidazolinone, triazolopyrimidine, pyr-imidinyl-thiobenzoates, and sulfonyl-aminocarbonyl-triazolinone) belongs to five chemical families of *ALS* inhibitors in *O. sativa* [206]. A novel allele (*G628W*) was developed from a G-to-T transversion at position 1882 of the *OsALS* gene and conferred resistance to herbicide stress. These mutants of rice plants conferred resistance to herbicides of imazethapyr (IMT) and imazapic (IMP) [209]. The CRISPR/Cas9 system was also successfully used to edit the *ALS1* gene of *G. max* to obtain chlorsulfuron-resistant plants [207]. Mutation of the Proline-186 residue in the *ALS* gene conferred chlorsulfuron resistance in *L. esculentum* [210]. An enzyme of activation-induced cytidine deaminase (AID) converts C to U in DNA/RNA by deamination. A synthetic complex of nuclease-deficient Cas9 fused to an AID, which is target-AID enables targeted nucleotide substitutions (C to T or G to A). The point mutation C287T of the *ALS* gene in rice plants resisted the herbicide imazamox [211]. In *T. aestivum*, herbicide-tolerant plants were produced by nucleotide editing of the acetolactate synthase (*ALS*) gene and acetyl-coenzyme A carboxylase gene, which conferred resistance to sulfonylurea, imidazolinone-, and aryloxy phenoxy propionate-type herbicides [205]. Co-editing three copies of the *ALS* gene resulted in herbicide tolerance in *Saccharum* spp. [42].

Glyphosate is one of the well-known and broad-spectrum herbicides used in the weed management of resistant crops, such as *C. annum*, *G. max*, *O. sativa*, and *Z. mays*. Glyphosate inhibits the enzyme *EPSPS* (5-enolpyruvylshikimate-3-phosphate synthase), involved in the biosynthesis of aromatic amino acids and secondary metabolites. Site-specific gene replacements and insertions in the rice endogenous *EPSPS* gene resulted in glyphosate-resistant plants [212,213]. The CRISPR/Cas9 tool creates a structural variation (genomic duplication or inversion) in chromosomes. The resulting mutant developed through CRISPR/Cas technology showed the increased accumulation of transcripts of *CP12* and *Ubiquitin2* genes and the 10-fold upregulated expression of *HPPD* (4-hydroxyphenyl pyruvate dioxygenase) and *PPO1* (protoporphyrinogen oxidase) resulted in herbicide resistance without affecting the yield and other agronomically important traits in *O. sativa* [214]. CRISPR-Cas9 system was used to edit the target genes of herbicides (*ALS* and *EPSPS*) in *L. esculentum* cv. Micro-Tom [215]. Another herbicide resistance gene, Bentazon Sensitive Lethal (*BEL*), gives resistance to herbicides of bentazon and sulfonylurea in *O. sativa*. CRISPR/Cas9-based mutation of the *BEL* gene was evaluated in rice using the *Agrobacterium*-mediated stable transformation [216]. The efficiency of mutagenesis ranged from 2% to 16%, and the phenotypic analysis indicated that the mutated plant was sensitive to the herbicide bentazon. Precise editing of the endogenous α-tubulin homolog gene *OsTubA2* using CRIPSR-mediated adenine base editors at the T1981 site. The point mutation in the *OsTubA2* gene transformed the *O. sativa* cultivar into a herbicide (dinitroaniline) tolerant cultivar [217] (Table 3). These studies summarised the application of CRISPR/Cas-mediated editing of genes responsible for metal and herbicide stress tolerance in plants.

**Table 3:** Application of the CRISPR-based genome editing approach in tailoring abiotic stress tolerant plants.

Crops	Targeted Gene	Trait	References
*Arabidopsis thaliana*	*OST2*	Drought tolerance	[117]
*Arabidopsis thaliana*	*AVP1*	Drought tolerance	[3]
*Arabidopsis thaliana*	*MIR169a* and *MIR827a*	Drought tolerance	[118]
*Arabidopsis thaliana*	*HAT*	Drought tolerance	[119]
*Arabidopsis thaliana*	*TRE1*	Drought tolerance	[120]
*Arabidopsis thaliana*	*NAC07*, *NAC019*, *NAC055*	Drought tolerance	[121]
*Arabidopsis thaliana*	*AITR3* and *AITR4*	Drought and salinity tolerance	[7]
*Arabidopsis thaliana*	*ACQO*	Salinity tolerance	[141]
*Arabidopsis thaliana*	*Oxp1*	Metal Stress tolerance	[13]
*Brassica napus*	*BnaA6.RGA*	Drought tolerance	[40]
*Glycine max*	*AITR*	Salinity tolerance	[41]
*Glycine max*	*SOS1*	Salinity tolerance	[139]
*Glycine max*	*ALS1*	Resistance to chlorsulfuron herbicide	[207]
*Hordeum vulgare*	*ITPK1*	Salinity tolerance	[134]
*Lactuca sativa*	*NCED4*	Heat tolerance	[179]
*Lycopersicon esculentum*	*SlLBD40*	Drought tolerance	[127]
*Lycopersicon esculentum*	*SlMAPK3*	Drought tolerance	[128,129]
*Lycopersicon esculentum*	*SlHyPRP1*	Salinity tolerance	[37]
*Lycopersicon esculentum*	*SlARF4*	Drought and salinity tolerance	[172]
*Lycopersicon esculentum*	*SlCBF1*	Cold tolerance	[186]
*Lycopersicon esculentum*	*SIAGL6*	Heat tolerance	[174]
*Lycopersicon esculentum*	*CPK28, APX2*	Heat tolerance	[175]
*Lycopersicon esculentum*	*BZR1*	Heat tolerance	[176]
*Lycopersicon esculentum*	*ALS*	Resistance to chlorsulfuron herbicide	[210]
*Oryza sativa*	*SRL1*, *SRL2*	Drought tolerance	[133]
*Oryza sativa*	*OsDST*	Drought and salinity tolerance	[130]
*Oryza sativa*	*OsERA1*	Drought tolerance	[132]
*Oryza sativa*	*SAPK2*	Drought and salinity tolerance	[131]
*Oryza sativa*	*RR22*	Salinity tolerance	[159]
*Oryza sativa*	*miR535*	Drought and salinity tolerance	[166]
*Oryza sativa*	*RAV2*	Salinity tolerance	[163]
*Oryza sativa*	*RR9*, *RR10*	Salinity tolerance	[171]
*Oryza sativa*	*NAC006*	Drought and heat tolerance	[180]
*Oryza sativa*	*OTS1*	Salinity tolerance	[168]
*Oryza sativa*	*HSP*	Heat tolerance	[173]
*Oryza sativa*	*HSA1*	Heat tolerance	[177]
*Oryza sativa*	*MYB30*	Cold tolerance	[190]
*Oryza sativa*	*Ann3*	Cold tolerance	[188]
*Oryza sativa*	*PRP1*	Cold tolerance	[192]
*Oryza sativa*	*WSL5*	Cold tolerance	[193,194]
*Oryza sativa*	*HAK1*	Low cesium accumulation	[199]
*Oryza sativa*	*LCT1,* *Nramp5*	Reduced cadmium accumulation	[198]
*Oryza sativa*	*NRAMP1*	Reduced levels of heavy metals (Cd and Pb)	[14]
*Oryza sativa*	*PRX2*	Potassium deficiency tolerance	[200]
*Oryza sativa*	*ARM1*	Increase tolerance to high Arsenic	[201]
*Oryza sativa*	*ALS*	Resistance to Imazethapyr and imazapic herbicides	[209]
*Oryza sativa*	*ALS*	Herbicide resistance	[204]
*Oryza sativa*	*ALS1*	Resistance to bispyribac-sodium herbicide	[208]
*Oryza sativa*	*ALS*	Resistance to Sulfonylurea, imidazolinone, triazolopyrimidine, pyr-imidinyl-thiobenzoates and sulfonyl-aminocarbonyl-triazolinone herbicides	[206]
*Oryza sativa*	*EPSPS*	Resistance to glyphosate resistance	[212]
*Oryza sativa*	*C287T*	Resistance to imazamox herbicide	[211]
*Oryza sativa*	*ALS*, *EPSPS*	Herbicide resistance	[215]
*Oryza sativa*	*BEL*	Resistance to bentazon herbicide	[216]
*Oryza sativa*	*OsTubA2*	Resistance to dinitroaniline herbicide	[217]
*Oryza sativa*	*Osbhlh024*	Salinity tolerance	[144]
*Oryza sativa*	*OsDERF1*	Drought tolerance	[116]
*Triticum aestivum*	*DREB1A/CBF3*	Drought tolerance	[122]
*Triticum aestivum*	*DREB2*, *ERF3*	Drought tolerance	[123]
*Triticum aestivum*	*HAG1*	Salinity tolerance	[143]
*Zea mays*	*ARGOS8*	Drought tolerance	[135]
*Zea mays*	*HKTI*	Salinity tolerance	[150]
*Zea mays*	*TMS5*	Heat tolerance	[178]
*Zea mays*	*ALS2*	Resistance to chlorsulfuron herbicide	[203]

## 6. Conclusions

The CRISPR/Cas genome editing tool provides a unique and innovative approach to producing crops with enhanced tolerance to environmental stress. It is considered the best genome editing method compared to ZFNs and TALENs due to its simplicity, low cost, and high efficiency. CRISPR/Cas can be used for gene knockout, knockdown, point mutation, replacement, and insertion to develop abiotic stress-tolerant plants. Multiplex genome editing systems can edit a single gene or a few genes, but editing polyploid crops is challenging because their entire genomes have been duplicated or triplicated. The availability of plant genome sequences allows researchers to tailor the genome precisely, facilitating the use of CRISPR/Cas9 in resistance breeding. However, some limitations are associated with this CRISPR/Cas system, such as off-target, which can be reduced by carefully designing sgRNAs and using specific nucleases. Also, PAM sequences provide target specificity and a guide to the genome editing sites. The application of the CRISPR/Cas system will increase with the development of the PAM-independent CRISPR/Cas systems in crop improvement. Numerous studies demonstrate that Cas enzyme modifications may decrease the PAM requirement and create a novel tool for gene functional studies. Several efficient computational tools, including CRISPR-GE, CRISPR-P, and CRISPR-PLANT-V2, have been developed to design the guide RNA precisely.

Genetic modification can integrate with efficient plant transformation and regeneration systems to attain the desired improvement. Efficient and robust transformation techniques are essential for introducing the CRISPR-Cas system into plant cells. Several methods, including *Agrobacterium*-mediated transformation or particle bombardment, are used for genetic manipulation, but many crops are recalcitrant or exceptionally hard to transform. In order to build an effective tissue culture and plant regeneration system, an attempt has been made to culture immature embryos that can transform into a complete plant. The commercialization and regulation of genome-edited crops are highly debated and vary across countries. Moreover, the generation of transgene-free crops takes longer due to several repeated back-crossings of the edited plant. The development of a DNA-free CRISPR/Cas system is expanding the usefulness and effectiveness of genome editing technology. However, the DNA-free genome editing technique is still evolving, and innovative strategies are urgently required to effectively implement in crop improvement.

The CRISPR/Cas-based genome editing technology presents excellent potential for fundamental and applied research, becoming a sustainable tool for resolving important biological and agricultural problems. The fundamental areas of plant research and improvement will depend on accurate information about the various aspects of the CRISPR/Cas9 system, such as the high-throughput genome sequencing method, guide RNA designing tools, and plant cell transformation and regeneration protocols.

## Figures and Tables

**Table 1:** Type of protospacer adjacent motifs (PAMs) sequences used in CRISPR/Cas genome editing system (adapted from [96]).

Name	Cas	Resources	PAM Sequence	PAM Location	Reference
SpCas9	Cas9	*Streptococcus pyogenes*	NGG	3′	[84]
St1Cas9	Cas9	*Streptococcus thermophilus*	NNAGAAW or	3′	[86]
			NGGNG		
SaCas9	Cas9	*Streptococcus aureus*	NNGRRT	3′	[87]
NmCas9	Cas9	*Neisseria meningitidis*	NNNNGATT	3′	[97]
FnCas9	Cas9	*Francisella novicida*	NGG	3′	[98]
CjCas9	Cas9	*Campylobacter jejuni*	NNNNRYAC	3′	[99]
AsCas12a	Cas12a(cpf1)	*Acidaminococcus* sp.	TTTV	5′	[25]
LbCas12a	Cas12a(cpf1)	*Lachnospiraceae bacterium*	TTTV	5′	[25]
FnCas12a	Cas12a(cpf1)	*Francisella novicida*	TTTN or YTN	5′	[25]
LsCas13	Cas13(C2c2)	*Leptotrichia shahii*			[100]
Cas14	Cas14	Archaea			[101]
FnCas9 variant	Cas9	Modified FnCas9	YG	3′	[98]
Modified SpCas9	Cas9	Engineered SpCas9	NGA or NAG	3′	[102]
SaCas9-KKH	Cas9	Engineered SaCas9	NNNRRT	3′	[88]
SpCas9-HF	Cas9	Engineered SpCas9	NGG	3′	[89]
eSpCas9	Cas9	Engineered SpCas9	NGG	3′	[90]
SpCas9-NG	Cas9	Engineered SpCas9	NG	3′	[85]
Sniper-Cas9	Cas9	Engineered SpCas9	NGG	3′	[91]
evoCas9	Cas9	Mutated SpCas9	NGG	3′	[92]
HypaCas9	Cas9	Mutated SpCas9-HF	NGG	3′	[93]
Cas9-NRNH	Cas9	Engineered SpCas9	NRNH	3′	[94]
SpG	Cas9	Engineered SpCas9	NGN	3′	[95]
SpRY	Cas9	Engineered SpCas9	NRN or NYN	3′	[95]

“N” is any nucleotide [“A”, “T”, “G’, “C”]. “R” is “A” or “G”. “H” is “A”, “C” or “T”. “Y” is “C” or “T”. “W” is “A” or “T”. Cas13 targets RNA sequences instead of DNA; Cas14 targets single-stranded DNA (ssDNAs) instead of double-stranded DNA (dsDNAs) and does not require a Protospacer-Adjacent Motif (PAM). Note: Adapted from Zhang et al. [96].

**Table 2:** Computational tools for designing guide RNA 9 (sgRNA).

Tool	Organism	Major Feature	Weblink
CHOPCHOP	>100 species, including plants	Provides several predictive models and primers. Visualizing the genomic location of genes and targets [103].	https://chopchop.cbu.uib.no/, accessed on 23 August 2023
Cas-OFFinder	>100 species, including plants	Searches potential off-target sites [104].	http://www.rgenome.net/cas-offinder/, accessed on 23 August 2023
CCTop	>100 species	Predictes off-target impacts and sgRNA efficiency using CRISPRater with custom in vitro transcription. Searching for single and multiple queries [105].	https://cctop.cos.uni-heidelberg.de/, accessed on 23 August 2023
CRISTA	>100 species	Detectes off-target, providing machine learning framework, including DNA/RNA genomic information and RNA thermodynamics [106].	https://crista.tau.ac.il/, accessed on 23 August 2023
CRISPR-GE	>40 plant species	PCR sequencing result analysis. Provides software toolkits, primer design, and on-target amplification [107].	http://skl.scau.edu.cn/, accessed on 23 August 2023
CRISPR-P	49 plant species	Providing on-target and off-target scoring and gRNA sequence analysis [108]	http://crispr.hzau.edu.cn/CRISPR2/, accessed on 23 August 2023
CRISPR-PLANT V2	7 plant species	Allows selection of particular chromosomes and a resource for specific gRNA spacer sequences [109].	http://omap.org/crispr2/, accessed on 23 August 2023
CRISPRlnc	10 species	Provides hundreds of lncRNAs and thousands of validated sgRNA [110].	http://www.crisprlnc.org/, accessed on 23 August 2023
SNP-CRISPR	9 plants and animal species	Designing sgRNAs (NGG and NAG) for targeting SNPs or Indels [111].	https://www.flyrnai.org/tools/snp_crispr/web/, accessed on 23 August 2023
PnB Designer	*O. sativa*, *V. vinifera*	Designing sgRNAs for base editors and pegRNAs for prime editors [112].	https://fgcz-shiny.uzh.ch/PnBDesigner/, accessed on 23 August 2023

## Data Availability

Not applicable.
